# Tracking the embryonic stem cell transition from ground state pluripotency

**DOI:** 10.1242/dev.142711

**Published:** 2017-04-01

**Authors:** Tüzer Kalkan, Nelly Olova, Mila Roode, Carla Mulas, Heather J. Lee, Isabelle Nett, Hendrik Marks, Rachael Walker, Hendrik G. Stunnenberg, Kathryn S. Lilley, Jennifer Nichols, Wolf Reik, Paul Bertone, Austin Smith

**Affiliations:** 1 Wellcome Trust-Medical Research Council Cambridge Stem Cell Institute, Cambridge CB2 1QR, UK; 2 Babraham Institute, Cambridge CB22 3AT, UK; 3 Wellcome Trust Sanger Institute, Hinxton CB10 1SA, UK; 4 Radboud University, Faculty of Science, Department of Molecular Biology, Radboud Institute for Molecular Life Sciences (RIMLS), Nijmegen 6500HB, The Netherlands; 5 Department of Biochemistry, University of Cambridge, Cambridge CB2 1GA, UK; 6 The Cambridge Centre for Proteomics, Cambridge System Biology Centre, University of Cambridge, Cambridge CB2 1QR, UK; 7 Department of Physiology, Development and Neuroscience, University of Cambridge, Cambridge CB2 4BG, UK; 8 Centre for Trophoblast Research, University of Cambridge, Cambridge CB2 3EG, UK

**Keywords:** ES cells, Rex1, Epiblast, Methylome, Pluripotency, Transcriptome

## Abstract

Mouse embryonic stem (ES) cells are locked into self-renewal by shielding from inductive cues. Release from this ground state in minimal conditions offers a system for delineating developmental progression from naïve pluripotency. Here, we examine the initial transition process. The ES cell population behaves asynchronously. We therefore exploited a short-half-life *Rex1::GFP* reporter to isolate cells either side of exit from naïve status. Extinction of ES cell identity in single cells is acute. It occurs only after near-complete elimination of naïve pluripotency factors, but precedes appearance of lineage specification markers. Cells newly departed from the ES cell state display features of early post-implantation epiblast and are distinct from primed epiblast. They also exhibit a genome-wide increase in DNA methylation, intermediate between early and late epiblast. These findings are consistent with the proposition that naïve cells transition to a distinct formative phase of pluripotency preparatory to lineage priming.

## Abstract (precis)

**Summary:** Extinction of mouse embryonic stem cell identity upon differentiation immediately follows collapse of the naïve pluripotency transcription factor circuitry and precedes upregulation of lineage-specific factors.

## INTRODUCTION

Epiblast cells, founders of all somatic cells and the germ line, are formed in the inner cell mass (ICM) on the final day of pre-implantation development in mice (Boroviak and Nichols, 2014; Cockburn and Rossant, 2010). This emergent condition of ‘naïve pluripotency’ (Nichols and Smith, 2009) is characterised by a unique suite of transcription factors, a hypomethylated genome, and the ability to give rise directly and clonally to embryonic stem (ES) cells (Boroviak et al., 2014; Brook and Gardner, 1997; Smith et al., 2012; Lee et al., 2014; Nichols and Smith, 2012). Upon implantation, ES cell-forming capacity is abruptly lost, epithelialisation commences, global gene expression is reconfigured and DNA methylation increases, which is indicative of a profound cellular transition (Boroviak et al., 2014, 2015; Auclair et al., 2014; Bedzhov and Zernicka-Goetz, 2014). Subsequently, egg cylinder epiblast cells are subject to inductive cues leading up to gastrulation and they become fated, although not yet lineage committed (Tam and Zhou, 1996; Osorno et al., 2012; Solter et al., 1970). The late phase of pluripotency during gastrulation is termed ‘primed’, reflecting the incipient expression of lineage-specification factors (Nichols and Smith, 2009; Hackett and Surani, 2014).

Mouse ES cells cultured in serum-free media supplemented with two chemical inhibitors (2i) of MEK1/2 and GSK3α/β, with optional addition of the cytokine LIF, are in a uniform condition of self-renewal termed the ‘ground state’ (Ying et al., 2008). In this *in vitro* ground state, ES cells show transcriptional and epigenetic similarity to naïve pre-implantation epiblast (Ficz et al., 2013; Habibi et al., 2013; Leitch et al., 2013; Nichols and Smith, 2012; Boroviak et al., 2015). Upon withdrawal of 2i, ES cells embark on a path to lineage commitment either *in vitro* or *in vivo* when injected into a pre-implantation embryo (Ying et al., 2008; Dunn et al., 2014; Marks et al., 2012). Recent studies have begun to explore the dissolution of naïve pluripotency and the route towards multi-lineage differentiation *in vitro* (Buecker et al., 2014; Leeb et al., 2014; Kurimoto et al., 2015; Thomson et al., 2011; Respuela et al., 2016; Yang et al., 2014; Betschinger et al., 2013; Davies et al., 2013; Liu et al., 2015; Acampora et al., 2013). However, differentiating cultures become heterogeneous (Marks et al., 2012; Kalkan and Smith, 2014; Buecker et al., 2014; Hayashi et al., 2011). A means to identify and select cells as they transition from naïve pluripotency would facilitate experimental resolution.

We previously generated ES cells carrying a *Rex1::GFPd2* (RGd2) reporter in which the coding sequence of one allele of Rex1 (gene name *Zfp42*) is replaced with a GFPd2-IRES-*bsd* cassette that produces a destabilised version of GFP protein with a 2-hour half-life (GFPd2) (Wray et al., 2011). Here, we exploit this reporter to monitor ES cell exit from naïve pluripotency guided by autocrine cues in defined adherent culture. We test the utility of the reporter as a faithful marker of naïve pluripotency and survey transcriptomic, metabolic and DNA methylome changes during the initial transition towards differentiation competence.

## RESULTS

### The RGd2 reporter is a neutral marker of naïve pluripotency in the embryo

The Rex1-coding sequence is entirely deleted in the *RGd2* allele. RGd2 ES cells (Wray et al., 2011) were transmitted through the mouse germline and heterozygous animals were backcrossed twice to strain 129. Following heterozygous intercrosses, homozygous mice were healthy and fertile, although slightly under-represented (Table S1). These results confirm previous reports that Rex1 is dispensable for development (Kim et al., 2011; Masui et al., 2008; Rezende et al., 2011). We could derive wild-type, heterozygous and homozygous ES cells, both male and female, from intercross embryos (Table S2), demonstrating that Rex1 is not significant for ES cell propagation. RGd2 expression should therefore constitute a neutral reporter.

**Fig. 1.**
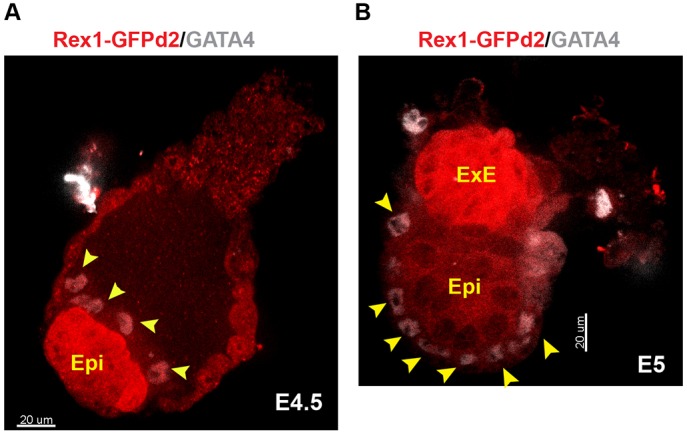
**Expression of the RGd2 reporter before and after implantation.** (A,B) Immunofluorescent staining for GFP (Rex1GFPd2) (red) and Gata4 (grey) at (A) E4.5 and (B) E5. Arrowheads show GATA4-positive nuclei. Scale bar: 20 μm. ExE, extra-embryonic ectoderm; Epi, epiblast.

We evaluated reporter expression in the embryo by immunofluorescence staining for GFP. Co-staining for GATA4 revealed that the RGd2 reporter is expressed exclusively and uniformly throughout the naïve epiblast (Epi) at E4.5 (Fig. 1A), with no GFP in either GATA4-positive primitive endoderm or trophoblast. GFP is downregulated during implantation and becomes undetectable in the epiblast at E5. However, expression is upregulated in the extra-embryonic ectoderm (ExE) (Fig. 1B). These results are consistent with Rex1 mRNA expression in the embryo measured by *in situ* RNA hybridisation (Pelton et al., 2002), RT-qPCR (Boroviak et al., 2014) and RNA-seq (Boroviak et al., 2015). We conclude that the *RGd2* allele faithfully reports endogenous *Rex1* transcription and accordingly that GFP expression coincides with naïve pluripotency *in vivo* (Boroviak et al., 2014).

### Release of ES cells from 2i triggers progression towards multi-lineage specification

**Fig. 2.**
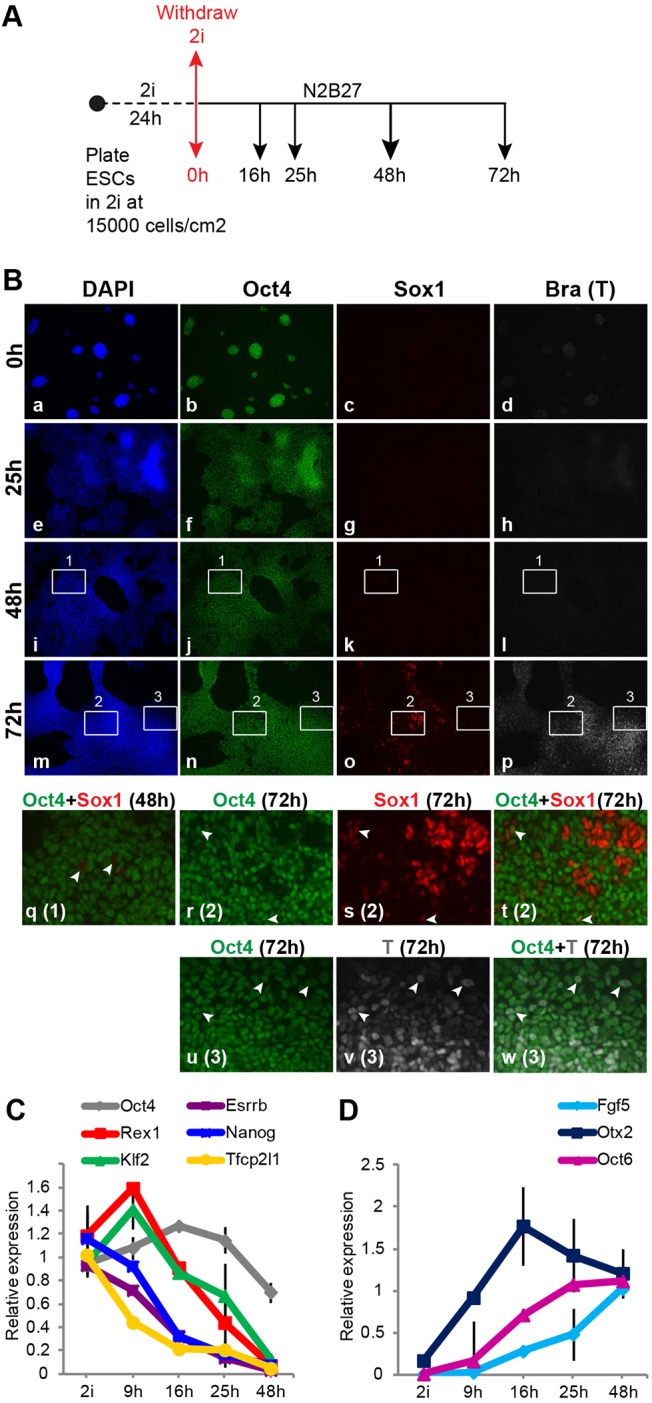
**Multilineage specification of ES cells upon release from 2i.** (A) Protocol for monolayer differentiation of naïve ES cells in N2B27 by withdrawal of 2i. (B) Immunofluorescent staining for Oct4, Sox1 and brachyury (T). Lower panels (q-w) show enlarged insets from 48 h and 72 h, with respective inset number in parentheses. (C,D) RT-qPCR for selected (C) pluripotency and (D) early post-implantation epiblast markers. Expression levels are shown as fold change relative to naïve ES cells in C and to 48 h samples in D. GAPDH was used for normalisation. Error bars indicate s.d. from two biological replicates.

We monitored the early phase of ES cell transition *in vitro* after withdrawal from 2i in serum-free N2B27 medium on gelatin-coated plastic (Fig. 2A). We started from ES cells in 2i alone because LIF delays the onset of differentiation (Dunn et al., 2014). Plating ES cells directly in N2B27 at low density (<10,000 cells cm^−2^) results primarily in neural differentiation (Ying et al., 2003). However, when cells were plated at an intermediate density (15,000 cells cm^−2^) and maintained in 2i for 24 h prior to withdrawal, numerous brachyury (*T*) -positive cells also appeared, indicative of non-neural specification (Fig. 2B). The latter conditions were used throughout this study.

Oct4 protein expression did not change substantially for 48 h after release from 2i (Fig. 2B). Rare cells expressing low levels of Sox1 were first detected at 48 h (Fig. 2B; panels k,q). By 72 h, clusters of bright Sox1-positive cells that lacked Oct4 emerged (Fig. 2B, panels o,r-t). Occasional Sox1/Oct4-double-positive cells were found outside these clusters (Fig. 2B; arrowheads in r-t). T-expressing cells were first detected at 72 h, mostly in dense clumps that were mutually exclusive with Sox1-positive clusters (Fig. 2B, m-p). T-positive cells at this stage were also positive for Oct4 (Fig. 2B; arrowheads in u-w), consistent with transient co-expression in early primitive streak and during directed *in vitro* differentiation (Thomson et al., 2011; Hoffman et al., 2013).

Oct4 mRNA downregulation started after 25 h (Fig. 2C). By contrast, transcripts for naïve transcription factors (TFs) (Dunn et al., 2014; Martello and Smith, 2014) declined within the first 25 h. Downregulation of Nanog, Esrrb and Tfcp2l1 initiated before Rex1 and Klf2. Concurrently, transcripts for early post-implantation epiblast markers Fgf5, Otx2 and Oct6 (*Pou3f1*) (Acampora et al., 2016; Pelton et al., 2002) were upregulated (Fig. 2D). mRNAs for naïve TFs were eliminated by 48 h (Fig. 2C). Similar results were observed with multiple ES cell lines (Fig. S1A,B).

These results indicate that, upon release from self-renewal in defined conditions, ES cells are driven by autocrine signals to progress from the naïve state to multi-lineage specification in an orderly sequence. First, naïve TFs are extinguished and markers diagnostic of post-implantation epiblast are induced. Subsequently, lineage-specific markers emerge and Oct4 is downregulated.

### Pluripotency factors display individual downregulation kinetics upon 2i withdrawal

**Fig. 3.**
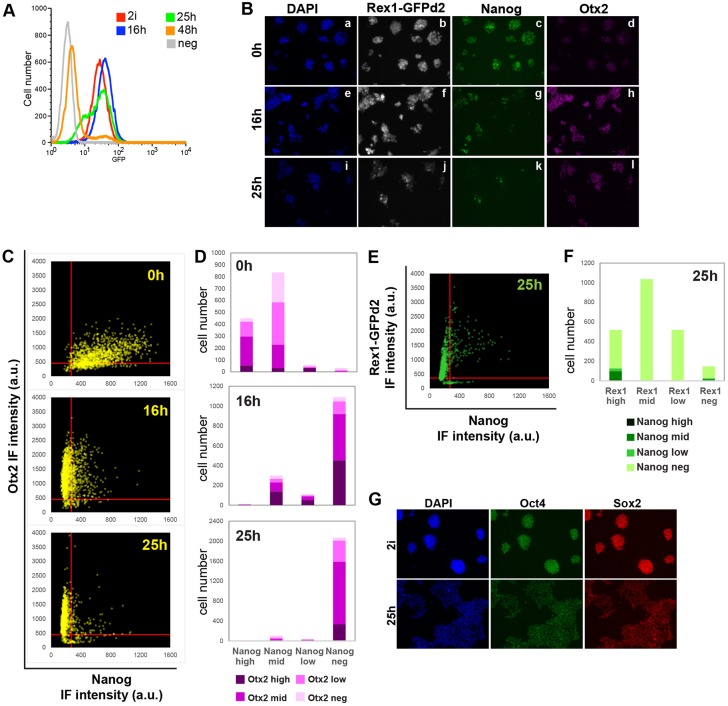
**Expression of transcription factors during transition of ES cells.** (A) GFP flow cytometry profile at indicated time points post-2i withdrawal (10,000 cells per time-point). Wild-type ES cells were used as the negative control (neg). (B) Immunofluorescent staining for GFP, Nanog and Otx2. (C) Otx2 versus Nanog fluorescence intensity per cell in arbitrary units (a.u.), as quantified by Volocity. X and Y intercepts of the red lines mark the cut-off for Nanog- and Otx2-negative cells, respectively. (D) Distribution of Otx2 expression in Nanog subpopulations. (E) GFP versus Nanog fluorescence intensity per cell. X and Y intercepts of the red lines mark the cut-off for Nanog- and GFP-negative cells, respectively. (F) Distribution of Nanog expression in Rex1 subpopulations at 25 h. (G) Immunofluorescent staining for Oct4 and Sox2.

To follow the kinetics of transition following release from 2i, we monitored the RGd2 reporter by flow cytometry (Fig. 3A). GFP was expressed unimodally with a log normal distribution in 2i. This tight peak persisted throughout the first 16 h after withdrawal, although mean fluorescence rose slightly, possibly owing to a transient increase in Rex1 mRNA at earlier time points (Fig. 2C, Fig. S1A). By 25 h, GFP intensity became heterogeneous with many cells shifted to lower expression. This profile suggests that Rex1 is downregulated with different kinetics in individual cells. By 48 h, the majority of cells extinguished GFP. Treatment with the protein synthesis inhibitor cycloheximide confirmed that the half-life of GFPd2 is slightly under 2 h in both 2i- and N2B27-cultured ES cells (Fig. S2A-C). Therefore, observed changes in GFP levels upon 2i withdrawal should track Rex1 transcription.

We compared the expression of the RGd2 reporter with the naïve TF Nanog and with Otx2, a TF that is upregulated in the peri-implantation epiblast (Acampora et al., 2013, 2016) (Fig. 3B). Quantification of fluorescence intensities for Nanog and Otx2 in single cells across the 25 h time course showed that in 2i almost all cells expressed Nanog at high or intermediate (mid) levels (Fig. 3C,D). Otx2 was expressed at low to intermediate (mid) levels in many cells, but was absent in 23% of cells. Sixteen hours post-2i withdrawal, GFP remained ubiquitous, Nanog became undetectable in most cells (72%) and Otx2 was upregulated (Fig. 3B-D). Nanog and Otx2 were co-expressed at mid or high levels in only 15% of cells. By 25 h, GFP intensity was heterogeneous, consistent with the flow cytometry profile. Otx2 was expressed in almost all cells, although the proportion of Otx2-high cells was lower than at 16 h, consistent with a relative decrease in Otx2 transcript levels (Fig. 2D). Nanog persisted in only 7% of cells (Fig. 3D), most of which were in the Rex1-high fraction (Fig. 3E,F). The overall pattern of Nanog and Otx2 expression during ES cell progression mirrors dynamics in the embryo. Nanog is co-expressed with low levels of Otx2 in the naïve pre-implantation epiblast, but as Nanog is downregulated in the peri-implantation epiblast, Otx2 is upregulated in a mutually exclusive fashion (Acampora et al., 2016).

A second naïve pluripotency factor, Tfcp2l1 (Martello et al., 2013; Ye et al., 2013), was already undetectable in most of the population by 16 h (Fig. S2D), concomitant with a rapid decrease in transcripts (Fig. 2C). We also examined Tfe3, a bHLH transcription factor that supports naïve pluripotency (Betschinger et al., 2013). Nuclear levels of Tfe3 were also already reduced by 16 h relative to 2i (Fig. S2E,F,G), with Tfe3 becoming mostly cytoplasmic. By contrast, Oct4 and Sox2 proteins exhibited homogeneous expression throughout the first 25 h after 2i withdrawal (Fig. 3G).

These results reveal that TFs associated with pluripotency display individual expression behaviour as ES cells transition from the ground state. RGd2 downregulation appears to reflect aggregate loss of naïve TFs against a background of persistent Oct4 and Sox2 expression.

### Exit from the ground state occurs asynchronously

**Fig. 4.**
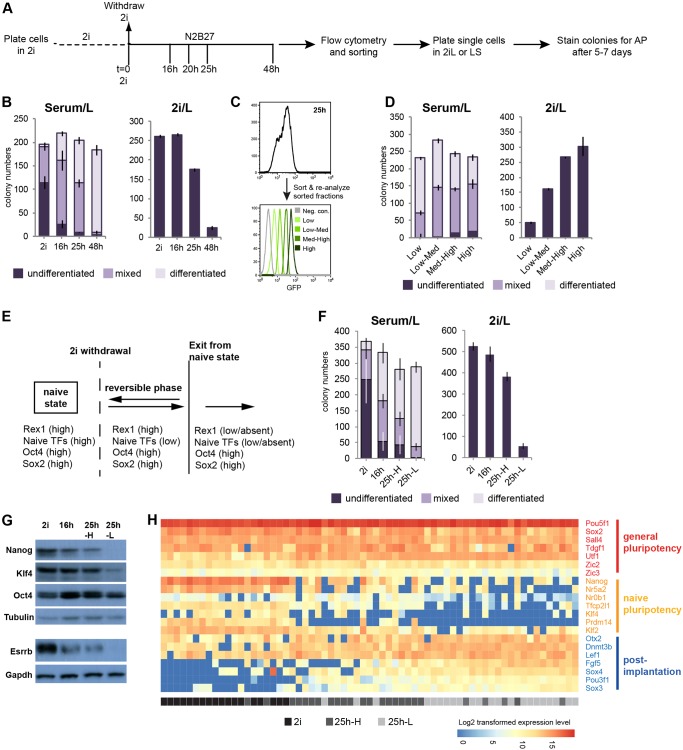
**Downregulation of Rex1 tracks exit from the naïve state.** (A) Protocol for sorting and clonogenicity assays. (B) Clonogenicity of cells from 2i and differentiating subpopulations sorted at indicated time points, plated in serum/LIF (Serum/L) or 2i supplemented with LIF (2i/L). Data are mean±s.d. from two technical replicates (500 cells were plated). (C) Sorting of 25 h-cultures into four subpopulations based on GFP levels by flow cytometry. Lower plot shows the GFP profiles of post-sort subpopulations. (D) Clonogenicity of four subpopulations shown in C. Data are mean±s.d. from two technical replicates. (E) Diagram summarising phases of transition from the naïve state. (F) Clonogenicity of the indicated subpopulations. Data are mean±s.d. from three biological replicates each with two technical replicates (800 cells were plated). (G) Immunoblot of total cell lysates from sorted subpopulations. β-Tubulin and Gapdh are loading controls. (H) Expression of selected general (red) and naïve (orange) pluripotency and early post-implantation epiblast (blue) markers in single cells measured using the Fluidigm system. Scale bar represents log2 transformed expression value.

To determine the time of exit from the ground state, entire cultures or subpopulations sorted on the basis of RGd2 expression at selected time points were re-plated at single cell density in serum/LIF (serum/L) and 2i/LIF (2i/L). Resulting colonies were stained for alkaline phosphatase (AP) activity (Fig. 4A).

Serum/L permits proliferation of both naïve ES cells and differentiating progeny (Marks et al., 2012). Thus, colonies in serum/L reflect plating efficiency and differentiation propensity. 2i-cultured ES cells and cells from inhibitor-withdrawn cultures generated similar numbers of colonies, indicating equivalent plating capacity (Fig. 4B). However, the proportions of colony types varied. From 2i cells, around 60% of colonies were wholly undifferentiated, with most of the remainder being mixed and a few completely differentiated. This heterogeneity is typical of ES cells plated in serum/L (Wray et al., 2010). The degree of differentiation increased with duration of 2i withdrawal; only 10% of colonies formed after 16 h were undifferentiated whereas over 20% were wholly differentiated. In 48 h cultures, 95% of colonies were differentiated (Wray et al., 2010). Thus, ES cells become increasingly predisposed to differentiation as the 2i withdrawal period is prolonged.

In 2i/L, self-renewal is optimal, but differentiating cells are eliminated and only naïve cells form colonies. Strikingly, the clonogenic efficiency of 16 h cells in 2i/L was equivalent to that of ground-state ES cells (Fig. 4B). Thus, the increased propensity for differentiation detected in serum/L is not matched by loss of self-renewal potential. However, after 25 h of 2i withdrawal, clonogenicity in 2i/L was significantly reduced, and by 48 h had fallen to 10% of the starting level. Therefore, up to 16 h after 2i withdrawal, self-renewal potential remains intact, despite the reduction in expression of some naïve TFs, induction of post-implantation epiblast markers and increased tendency to differentiate in serum/L. Between 16 h and 25 h, self-renewal capacity is partially lost, whereas by 48 h, exit from the naïve state is almost complete across the culture. Thus, exit from the naïve state proceeds gradually in the ES cell population over an extended period (≤48 h).

### Downregulation of Rex1 tracks loss of ES cell self-renewal potential

To determine whether the gradual loss of ES cell identity at the population level is recapitulated at the single cell level, we exploited flow cytometry to fractionate cells based on RGd2 expression. We sorted for subpopulations 25 h post-2i withdrawal and then replated (Fig. 4C,D). In serum/L, colony numbers were relatively constant, although the proportion of undifferentiated colonies declined with decreasing GFP. In 2i/L, marked differences in total colony numbers were evident (Fig. 4D). The GFP-high fraction exhibited equivalent clonogenicity to 2i cells (Fig. 4B,D), indicating complete retention of naïve status. However, subpopulations with lower GFP levels produced progressively fewer colonies. The number of colonies formed from the GFP-low fraction was only 15% of the number from 2i or GFP-high cells. Thus, the great majority of this subpopulation has departed the ES cell state (Fig. 4D). These data demonstrate that by 25 h the population has become functionally heterogeneous. Therefore, ES cells transition asynchronously.

To examine how closely exit from the naïve state and downregulation of Rex1 coincide, we sorted cultures 20 h post-2i withdrawal, when the first GFP-low cells appear, into GFP-high and GFP-low subpopulations using the same gates as for 25 h cultures (Fig. S3A). Clonogenic efficiency of GFP-high cells in 2i/L was equivalent to ground state ES cells, but was reduced by about 70% for GFP-low cells (Fig. 4B, Fig. S3A). Thus, the earliest cells that we could obtain after Rex1 downregulation have mostly exited the ES cell state. These data suggest that in individual cells the transition occurs at, or slightly after, loss of Rex1 expression.

We examined whether cell cycle dictates the kinetics of Rex1 downregulation. We stained ES cells with the DNA-binding dye Hoechst and isolated subpopulations in G1, S and G2/M by flow cytometry (Fig. S3B). We plated these cells along with stained but unsorted controls directly in N2B27 at 3×10^4^ cells cm^−2^, which approximates the density at the time of 2i withdrawal in our standard protocol. All populations displayed a similar heterogeneous GFP distribution 25 h after plating, although the G1 starting subpopulation showed a marginally narrower range and a slightly lower mean intensity (Fig. S3C). We conclude that the kinetics of Rex1 downregulation is largely independent of initial cell cycle phase.

For subsequent analyses, we selected and defined cell populations as follows: 2i- cells represent the ground state; 16 h and 25h-H cells are in a reversible phase preceding the extinction of ES cell character; and 25h-L cells are the primary products of exit from naïve pluripotency (Fig. 4E). Flow cytometry and colony assays confirmed the reproducibility of this system (Fig. 4F, Fig. S3D,E). Colonies formed from 16h and 25h-H populations after replating in 2i/L re-expressed naïve pluripotency markers at the same level as 2i cells, and downregulated Otx2, Oct6 and Fgf5 (Fig. S4A,B), demonstrating that the ground state was re-established. Immunoblotting after 2i withdrawal showed progressive downregulation of Nanog, Esrrb and Klf4 proteins and decreasing GFP, whereas Oct4 was constant (Fig. 4G). The difference between 25h-H and 25h-L cells is particularly interesting: Nanog and Esrrb proteins are almost undetectable in 25h-L cells and Klf4 is greatly diminished. These three factors are pivotal members of the ES cell gene regulatory circuitry (Dunn et al., 2014) and their elimination, together with the absence of Tfcp2l1 and nuclear Tfe3 (Fig. S2), is expected to be sufficient for elimination of self-renewal in 25h-L cells.

To assess further the variation between 25 h-cells, we performed single cell RT-qPCR for selected genes (Fig. 4H). This analysis confirmed that general pluripotency factors remained constant or showed modest changes, whereas naïve TFs and post-implantation markers in general showed reciprocal expression. Notably, 2i cells were devoid of Fgf5, Oct6 (Pou3f1), Sox3 and Sox4 transcripts that are upregulated in the post-implantation epiblast (Boroviak et al., 2015; Pelton et al., 2002; Acampora et al., 2016). The 25h-H cells showed variable upregulation of these four markers and downregulation of no more than three of the naïve TFs. By contrast, in 25h-L cells all the post-implantation epiblast markers were co-expressed and at least four of the naïve TFs were downregulated. These results suggest that decay of ES cell identity correlates with cumulative loss of naïve TFs and concomitant cumulative upregulation of factors associated with early post-implantation epiblast. In the reversible 25h-H population, these factors are expressed in various combinations without an evident hierarchy.

### Transcriptional changes during transition from naïve pluripotency

**Fig. 5.**
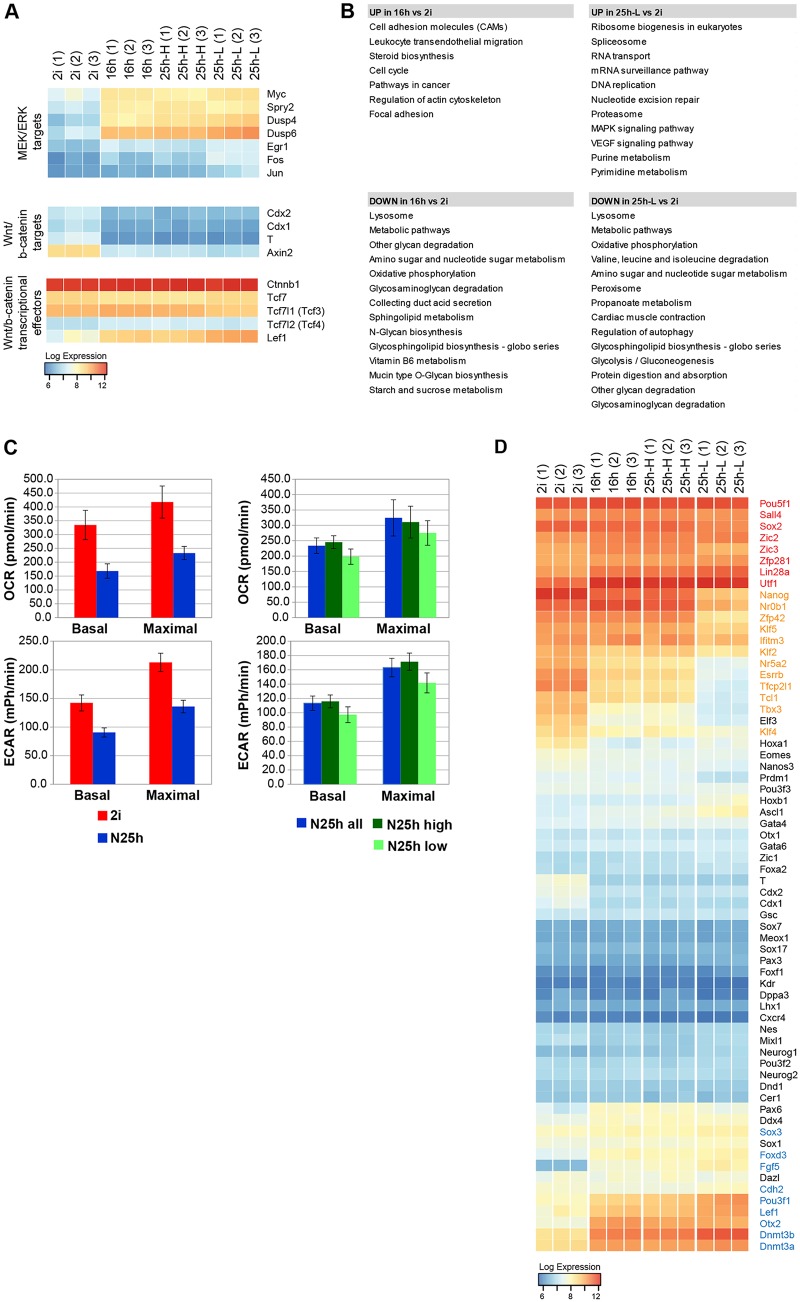
**Transcriptional changes in ES cells during progression from naïve pluripotency.** (A) Expression of MEK/ERK and Wnt/β-catenin transcriptional effectors and targets from three independent replicates measured by microarray profiling. Scale represents log2 transformed expression value. (B) Enriched KEGG pathway categories in the differentially expressed gene sets ranked according to *P* value (*P*<0.05). (C) OCR and ECAR levels of 2i versus 25 h populations (left) and sorted 25h-H and 25h-L subpopulations with unsorted whole population (right) (data are mean± s.d. from six technical replicates). (D) Expression of general (red) and naïve (orange) pluripotency, early post-implantation (blue) and lineage-priming factors (black) detected by microarray. Scale represents log2 transformed expression value.

To examine global expression dynamics, we carried out microarray profiling using three biological replicates. We also performed RNA-seq on independently derived RGd2 ES cell lines. We found a total of 8810 genes in the microarray that were differentially expressed between at least two subpopulations (Table S3). Consistent with the activation of MEK/ERK and GSK3 upon 2i withdrawal, we observed changes in the expression of components of the pathway and transcriptional targets. Activation of MEK/ERK is reflected in the upregulation of immediate ERK response genes, such as *Egr1*, *Fos*, *Myc* and *Jun* (Murphy et al., 2004), and of negative-feedback regulators *Spry2* and the ERK phosphatases *Dusp4* and *Dusp6* (Fig. 5A). mRNAs for the canonical Wnt targets *T*, *Axin2*, *Cdx1* and *Cdx2* are detected at low levels in 2i, consistent with inhibition of Gsk3 (Marucci et al., 2014) (Fig. 5A). Expression is reduced upon 2i withdrawal, indicating reduction of β-catenin-dependent transcription during transition from the ground state. Lef1 is upregulated, however, suggesting increased potential for Wnt-stimulated transcriptional regulation after exit.

KEGG pathway enrichment analysis revealed that highly upregulated genes in 16 h-cells were associated with cell cycle, cytoskeleton, steroid synthesis and cell adhesion; and in 25h-L cells with ribosome biogenesis, RNA processing, DNA replication, nucleotide metabolism, proteosome, VEGF signalling and MAPK pathway. Most downregulated in 16h- and 25h-low cells were genes with functions in lysosomes, oxidative phosphorylation (OxPhos), glycolysis, glycosylation and glycan degradation. (Fig. 5B). An overall decrease in transcripts encoding components of mitochondrial respiratory complexes was confirmed by RNA-seq (Fig. S5A). The changes encompassed all five mitochondrial enzyme complexes that mediate electron transport and ATP synthesis. To investigate metabolic consequences, we measured oxygen consumption rate (OCR) and extracellular acidification rate (ECAR), indicators of mitochondrial respiration and glycolysis, respectively. Naïve ES cells exhibited higher basal and maximal OCR and ECAR than the 25 h populations, indicating higher levels of both mitochondrial respiration and glycolysis (Fig. 5C). Within the 25 h population, 25h-H cells exhibited higher OCR and ECAR than 25h-L cells (Fig. 5C), indicating that the switch in metabolism is not a direct response to inhibitor withdrawal but is associated with a developmental transition. A reduction in mitochondrial respiration between naïve and primed pluripotent stem cells has been reported in mouse and human (Takashima et al., 2014; Zhou et al., 2012; Guo et al., 2016), and has also been proposed to occur *in utero* (Zhou et al., 2012). Our analyses indicate that metabolic resetting begins during transition from naïve pluripotency and initially involves reduction in both oxidative phosphorylation and glycolysis.

To benchmark developmental progression, we curated panels of markers for the following categories: general pluripotency, naïve pluripotency, post-implantation epiblast and lineage specification. We then examined expression in our microarray (Fig. 5D, Table S3) and RNA-seq (Fig. S5B,D, Table S4) datasets. Most naïve pluripotency TFs were downregulated in reversible cells, and almost absent in 25h-L cells, in accordance with decay of ES cell identity. Exceptions were Rex1, Klf5, Fbxo15 and Nr0b1, which were maintained in reversible cells and reduced but not eliminated in 25h-L cells (Fig. 5D, Fig. S5B). None of this latter group of factors is a component of the core gene regulatory circuitry of naïve pluripotency (Dunn et al., 2014). Among the general pluripotency markers, Oct4 and Sall4 remained constant, whereas Sox2 transcripts were reduced by 70% on average (Fig. S5B, Table S4) in 25h-L cells, similar to its downregulation in the epiblast from E4.5 to E5.5 (Boroviak et al., 2015). Other pluripotency factors, including Lin28a, Zfp281, Zic2 and Utf1 exhibited upregulation (Fig. 5D, Fig. S5B). Consistent with increased expression, Lin28a, Zfp281 and Zic2 are reported to drive transition from naïve to primed pluripotency (Zhang et al., 2016; Fidalgo et al., 2016; Luo et al., 2015; Betschinger et al., 2013).

To assess concordance with protein levels, we performed mass spectrometric analysis via stable isotope labelling of amino acids in culture (SILAC). These data confirmed that relative nuclear protein levels of TFs associated with naïve and general pluripotency correlated with respective transcripts, except for Rex1 and Klf5, levels of which were not reduced in 25h-L cells, despite decreasing transcript levels (Fig. S5C).

Factors that are upregulated in the post-implantation epiblast (Boroviak et al., 2015) [Lef1, Pou3f1 (Oct6), Dnmt3a/b, Foxd3, Sox3, Fgf5, Cdh2 and Otx2] were progressively induced upon 2i withdrawal (Fig. 5D, Fig. S5B). A large panel of factors associated with commitment to germ line, neuroectoderm, endoderm or mesoderm lineages remained at near-background levels (RPKM<10) and showed no upregulation beyond levels expressed in naïve ES cells (Fig. 5D, Fig. S5D). Of note, Gata4 and Gata6 were not induced in 25h-L cells, excluding primitive endoderm specification as an alternative path. These results establish that ES cell exit from naïve pluripotency is temporally segregated from upregulation of lineage determination factors.

### Comparison of ES cell progression with *in vivo* epiblast, EpiLCs and EpiSCs

**Fig. 6.**
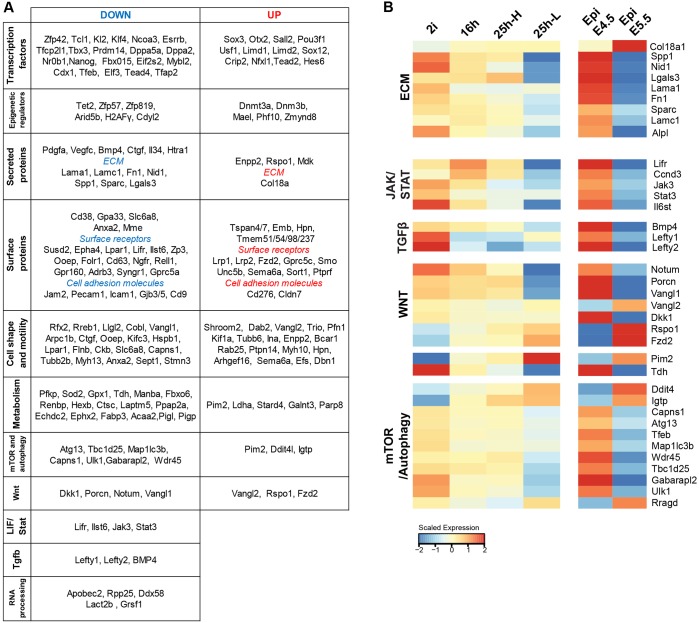
**Comparison of transcriptional changes during pluripotency progression in ES cells and in the embryo.** (A) Functional grouping of genes that show similar regulation in ES cells and in the embryo. (B) Expression of genes from selected pathways.

We compared the RNA-seq data from our *in vitro* populations (Table S4) with data from embryo samples acquired by a small-sample RNA-seq protocol (Boroviak et al., 2015). We isolated expression changes that occur between pre- and post-implantation epiblast (E4.5 and E5.5), and asked to what extent these are reflected in the *in vitro* transition. Out of 608 genes that are differentially expressed between E4.5 and E5.5 epiblast and robustly detected in one or both of the samples (FPKM≥10), more than half (366 of 608) exhibited differential expression during ES cell transition, with the direction of change conserved (Fig. 6, Table S5). Several functional groups could be identified within the shared up- and downregulated gene sets (Fig. 6A). Besides transcription factors and epigenetic regulators with established functions in the epiblast, the common group included genes associated with extracellular matrix (ECM), cell adhesion, motility, shape, metabolism and autophagy. Transcripts for ECM components, such as fibronectin (Fn1), laminin isoforms (Lamc1 and Lama1), the laminin-linker protein Nid1, Spp1 (osteopontin), Sparc, Lgals3 and Alpl were downregulated, whereas Col18a was upregulated (Fig. 6B), indicating major reconfiguration of ECM production. Tissue non-specific alkaline phosphatase (Alpl), which is widely used as a surrogate marker for ES cells, modifies the ECM by dephosphorylating ECM molecules such as osteopontin (Spp1) (Narisawa, 2015).

The LIF receptor components Lifr and Ilst6, the signal transducers Jak3 and Stat3, and the transcriptional targets Ccnd3, Klf4 and Tfcp2l1 were downregulated *in vitro* and *in vivo*, indicating that diminished LIF signalling is a common feature in transition from naïve pluripotency (Fig. 6B). BMP4 and the Nodal inhibitors Lefty1 and Lefty2 were also downregulated, together with altered expression of positive and negative regulators of Wnt signalling. These events highlight a changing signalling context.

We noted changes in enzymes that regulate metabolism and autophagy. Threonine dehydrogenase (Tdh) was downregulated. This is a vital enzyme for mouse ES cell survival that converts threonine into acetyl co-A and glycine, feeding the TCA cycle and purine synthesis (Wang et al., 2009). *Pim2*, one of the most highly upregulated genes in both settings (Fig. 6C), is a kinase that promotes glycolysis (Yu et al., 2013) and mTOR signalling (Lu et al., 2013; Zhang et al., 2015). The mTOR pathway inhibitor Ddit4 (Redd1), and the mTOR activator Rragd were both upregulated. mTOR signalling is activated during ES cell differentiation (Betschinger et al., 2013), and it has been reported that mTOR inhibition induces a diapause-like state of arrested development in the mouse blastocyst (Bulut-Karslioglu et al., 2016). Pim2, Ddit4 and Rragd are candidates that might contribute to complex mTOR regulation during this transitional period. Activated mTOR suppresses autophagy by phosphorylating and inhibiting Ulk1 and Atg13, two factors that are required for autophagy initiation, and through phosphorylation-dependent cytoplasmic sequestration of Tfeb, a TF that orchestrates the expression of genes involved in lysosome function and autophagy (Kim and Guan, 2015; Napolitano and Ballabio, 2016). These three mTOR targets, Ulk1, Atg13 and Tfeb, along with several autophagosome-associated factors were downregulated (Fig. 6B), suggesting transcriptional and post-transcriptional suppression of autophagy as the cells transition from naïve pluripotency *in vitro* and *in vivo*.

We found that 190 of the 608 genes that show differential expression during epiblast transition do not change their expression between 2i ES cells and 25h-L cells, whereas 52 showed differential expression in the opposite direction to the embryo (Table S5). This latter subset included the ERK target Egr1 and factors that regulate cell proliferation (Atf3, Tef, Trp53, Tada3, Klf6 and Ccng1), apoptosis (Apaf1 and Bid), cell adhesion and morphology (Krt18, Cdh2, Fez1, Lamb1, Tns3 and Amotl1), as well as signalling pathway components, such as Notch3, Rbpj and the Nodal co-receptor Tdgf1 (Cripto). Contrasting expression of these genes might reflect differences in the developmental snapshots sampled *in vitro* and *in vivo*, and/or the absence of paracrine signalling cues in our minimal culture system. Nonetheless, overall the transcriptome analyses support the idea that loss of Rex1 expression in a simple and defined ES cell culture system mimics several features of the developmental transition from pre- to post-implantation pluripotency.

We also compared 25h-L cells with post-implantation epiblast-like cells (EpiLCs), a transient intermediate generated during *in vitro* germ cell differentiation by plating ES cells from 2i/L into Fgf2, activin and 1% KSR for 48 h (Hayashi et al., 2011; Buecker et al., 2014). Differential gene expression analysis showed that EpiLCs were overall similar to 25h-L cells, and only 183 genes are differentially expressed between them (Table S6). We also generated EpiLCs from RGd2 ES cells and measured reporter expression by flow cytometry. We found that a subpopulation of EpiLCs (23%) expresses Rex1 at naïve ES cell levels (Fig. S6A,B), indicating that EpiLC populations are heterogeneous and contain a substantial fraction of undifferentiated ES cells.

We additionally undertook a comparison with published data from post-implantation epiblast-derived stem cells (EpiSCs), which are related to gastrula stage epiblast (Kojima et al., 2014). Marker expression (Fig. S6C,D) shows that 25h-L cells are related to E5.5 epiblast and are distinct from EpiSCs. These data confirm that ES cells do not transition directly into EpiSCs (Hayashi et al., 2011; Smith, 2017).

### Acquisition of DNA methylation during transition from naïve pluripotency

**Fig. 7.**
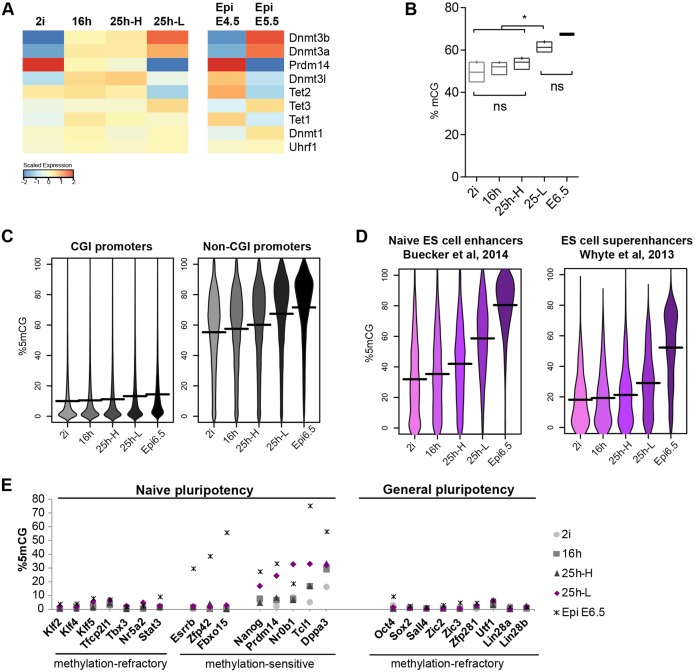
**Acquisition of DNA methylation during transition from naïve pluripotency.** (A) mRNA expression of factors that modulate DNA methylation. (B) Global genomic methylation in CG context (mCG) in 2 kb tiles. ns, non significant, *P<0.05 (one-way multiple comparisons ANOVA corrected with Tukey's test). (C,D) Percentage of mCG (C) in the promoters (−1000 bp to +500 bp of TSS) of expressed genes (RPKM≥10), (D) in genome-wide naïve and super enhancers and (E) in the promoters of selected pluripotency-associated genes.

Genome-wide DNA methylation increases substantially between E4.5 and E5.5 *in utero* (Auclair et al., 2014). Expression of *de novo* DNA methyltransferases Dnmt3a and Dnmt3b is upregulated both in ES cells and in the epiblast during transition (Figs 5D, 7A, Fig. S5B). By contrast, Prdm14, which represses Dnmt3a/b and promotes Tet activity on target genes (Yamaji et al., 2013; Okashita et al., 2014; Ficz et al., 2013), is downregulated. Whole-genome bisulfite sequencing (WGBS) revealed an increase in total CG methylation across the genome upon 2i withdrawal (Fig. 7B). Average genome methylation tended to increase in small increments between 2i, 16h and 25h-H, with a more pronounced and statistically significant increase at 25h-L. The increase was similar across gene bodies, exons, introns, intergenic regions, satellites and retrotransposon sequences (LINEs, SINEs, LTRs, IAPs), whereas methylation of CpG islands (CGIs) was not generally altered (Fig. S5A,B). Promoters that contain CGIs remained refractory to DNA methylation upon 2i withdrawal, whereas non-CGI promoters exhibited increased methylation similar to the genome average (Fig. 7C).

Whole-genome methylation data are not available for E5.5 epiblast. We therefore compared our profiles with published data on E6.5 post-implantation epiblast (Seisenberger et al., 2012). This analysis showed that the CG methylation level of 25h-L cells is intermediate between naïve ES cells and E6.5 post-implantation epiblast across all genomic regions (Fig. 7, Fig. S7). This is in line with reduced representation bisulfite sequencing (RRBS) data on embryos that shows a marked increase in methylation between E4.5 and E5.5 with a further increase at E6.5 (Auclair et al., 2014).

To investigate how DNA methylation might relate to gene expression changes during exit from the naïve state, we examined enhancers. We identified ‘naïve enhancers’ from published ChIP-seq datasets from naïve ES cells (Table S8) (Buecker et al., 2014) as regions displaying the general enhancer mark H3K4me1, together with the active enhancer marks H3K27Ac and p300 (Visel et al., 2009; Rada-Iglesias et al., 2011; Heintzman et al., 2007; Visel et al., 2013; Blow et al., 2010). In 2i, these enhancers were lowly methylated, but they gained methylation progressively upon 2i withdrawal (Fig. 7D). By contrast, ES cell ‘super enhancers’ (SEs) defined in serum-cultured ES cells (Whyte et al., 2013; Hnisz et al., 2013) exhibited low and relatively constant methylation levels in 2i, 16h and 25h-H cells, with a small increase in 25h-L cells but below the genome average (Fig. 7D). These observations indicate that, on exit from naïve pluripotency, naïve enhancers are methylated, which is indicative of decommissioning, whereas SEs that may be linked to general pluripotency-associated transcription are protected from methylation.

We compared our WGBS data with published RRBS data from E4.5 and E5.5 epiblasts (Auclair et al., 2014), and Rex1-sorted subpopulations of serum-cultured RGd2 ES cells (Singer et al., 2014) (Table S9). This analysis is limited to promoters and CGIs that are covered in all datasets, because RRBS enriches for genomic regions with high CpG content (Meissner et al., 2005). The vast majority of CGIs and CG-rich promoters in epiblast samples exhibited less than 5% methylation (Fig. S8A, Table S9). This was matched in 2i and 25h-L cells. By contrast both Rex1-high and Rex1-low fractions of serum-cultured ES cells exhibited higher levels of methylation. Therefore, we did not include serum ES cell samples in further analyses. Most promoters gained less than 5% methylation during ES cell and epiblast transitions (Fig. S8B). We asked whether those that do gain methylation are conserved between *in vivo* and *in vitro* settings. We isolated 2000 promoters with the highest methylation increases (Table S10). Interestingly, these promoters had higher methylation relative to all other promoters in naïve ES cells and epiblast (Fig. S8C). The majority were associated with lowly expressed or non-expressed genes (Table S10). In the common group of 1288 promoters were only 52 associated genes that are expressed in both in ES cell populations and the epiblast (Fig. S8D). A subset of these genes exhibited downregulation both in ES cell and epiblast progression. Among these are *Tdh*, *Lefty1*, *Tcl1* and *Prdm14*.

WGBS analysis also showed no genome-wide correlation between promoter methylation and gene expression changes. Nonetheless, we noted increased methylation in a subset of naïve pluripotency gene promoters, including *Nanog*, *Nr0b1* and *Dppa3*, in addition to *Prdm14* and *Tcl1*, as noted above (Fig. 7E, Fig. S8E). Promoters of other naïve and general pluripotency factors did not gain methylation, showing that pluripotency-associated genes acquire methylation with different kinetics. Thus, we conclude that promoter methylation is not a major driver of transcriptional changes during exit from the naïve state, although it might contribute to repression of a subset of genes that are of potential functional significance for the transition.

## DISCUSSION

The RGd2 reporter provides near real-time detection of exit from naïve pluripotency and enables isolation of the first cells to change functional identity. Loss of Rex1 expression marks a progression in pluripotent status that precedes a decline in Oct4 level or acquisition of lineage-specific gene expression. Our results characterise a defined *in vitro* monolayer differentiation system in the absence of uterine or extraembryonic cues. In these simple conditions autocrine signals are sufficient to drive transcriptomic, metabolic and methylome changes that are broadly convergent with peri-implantation epiblast. These findings indicate that the gene regulatory circuitry of ES cells has an innate capacity to orchestrate a profound developmental transition.

At the onset of this transition, the molecular network that sustains naïve pluripotency is dismantled (Buecker et al., 2014; Kalkan and Smith, 2014; Leeb et al., 2014). Apparently co-incident with acute downregulation of the critical naïve TFs, post-implantation epiblast markers are up-regulated (Acampora et al., 2016; Boroviak et al., 2015). Increased differentiation when transferred to serum suggests enhanced sensitivity to inductive cues before cells have fully extinguished ES cell identity. However, for as long as Rex1 is expressed, cells retain in full the ability to regain the ground state. Such reactivation of self-renewal, despite marked reduction in the levels of functionally important naïve TFs, is consistent with evidence that the mouse ES cell state is founded on a highly flexible transcription factor circuitry (Dunn et al., 2014; Martello and Smith, 2014; Young, 2011; Niwa, 2014). We surmise that loss of Rex1 reflects a cumulative reduction of the suite of factors below a critical threshold. From this point, the naïve TF network cannot be reactivated and is subsumed by an emerging new circuitry. The apparent gradual loss of self-renewal gleaned from whole population analyses arises from asynchronous single cell dynamics and at the level of individual cells the exit from ES cell identity may be precipitate.

Rex1 transcription is considered to be directly regulated by several naïve TFs (Chen et al., 2008; Kim et al., 2008) which can explain how the RGd2 reporter serves as a sensor of the overall activity of the naïve transcription factor circuitry. Nevertheless, 10-15% of the Rex1-low cells at 25 h can be restored to ground state self-renewal. This may be explained in part by incomplete efficiency of flow sorting, but also suggests that Rex1 downregulation might be separated from exit in a minority of cells. The higher incidence of reversion for Rex1-Low cells at 20 h is consistent with silencing of Rex1 slightly preceding loss of ES cell identity. Reversibility of Rex1 reporter expression has been reported in serum (Toyooka et al., 2008), where ES cells are continuously exposed to conflicting pro- and anti-differentiation stimuli that may perturb developmental progression. Even in those conditions, however, it is apparent that with more stringent categorisation of Rex1-negative cells, reversion frequency is low (Nakai-Futatsugi and Niwa, 2016). Furthermore, Rex1-negative cells from serum culture tend to be eliminated from blastocyst chimaeras, indicating limited identity with resident epiblast (Alexandrova et al., 2016; Toyooka et al., 2008). Nonetheless, the connection between down-regulation of naïve factors and loss of Rex1-GFP may not be invariant. Indeed, rare Nanog-positive/GFP-negative cells are detected at 25 h.

Consistent with loss of functional ES cell character, the 25h-L population show significant transcriptome variance from their naïve predecessors. They are clearly distinct from EpiSCs, and converge towards EpiLC and E5.5 epiblast. It will be of interest to determine to what extent micro-environmental modulations, such as substrate composition, may increase the veracity of the ES cell model.

The dynamic and global increase in DNA methylation as ES cells leave the naïve state generate a methylome intermediate between naïve and primed pluripotent compartments. We detected profound increases in the methylation levels of naïve ES cell enhancers and in a minority of promoters. However, there was no overall correlation between promoter methylation and gene expression, as also observed when ES cell cultures were switched between 2i and serum (Ficz et al., 2013; Habibi et al., 2013). Thus, increased promoter methylation does not appear to be a major driver of initial progression from naïve pluripotency. Indeed most pluripotency gene promoters were spared from methylation, although *Nanog* and *Prdm14* were prominent exceptions that gained methylation in 25h-L cells.

We have proposed that downregulation of naïve pluripotency factors elicits differentiation competence via an intermediate phase of “formative pluripotency” (Kalkan and Smith, 2014; Smith, in press). This is postulated as a period of competence acquisition for multi-lineage specification. *In vivo* the formative phase corresponds to peri- and immediate post-implantation epiblast (E4.75-5.75), before cells exhibit expression of lineage specification factors. Notably during this period epiblast cells acquire competence for germ cell induction (Ohinata et al., 2009; Hayashi et al., 2011). Our results indicate that ES cells that downregulate Rex1 and depart naïve pluripotency show transcriptome and methylome features that may be anticipated for immediate post-implantation epiblast. Thus, these Rex1-low cells represent a snapshot of the nascent formative phase, undergoing rewiring of the gene regulatory network and remodelling of the epigenome. The datasets we provide constitute a resource for inspecting an overlooked phase of pluripotency. It will be of future interest to dissect in detail the molecular dynamics and drivers of transition in this defined and simple system and also to determine whether the formative phase may be suspended as a stem cell state in culture, as achieved for naïve ES cells and primed EpiSCs.

## MATERIALS AND METHODS

### ES cell lines and culture

ES cell lines carrying the RGd2 reporter were derived from embryos using previously described protocols (Nichols et al., 2009). For routine maintenance, ES cells were plated at 2-3×10^4^ cells cm^−2^ in 2i on 0.1% gelatine-coated dishes (Sigma, G1890) and passaged every 3 days following dissociation with Accutase (PAA, L11-007). 2i consists of N2B27 (NDiff N2B27 base medium, Stem Cell Sciences, SCS-SF-NB-02) or lab-made N2B27, supplemented with PD0325901 (1 μM) and CHIR99021 (3 μM). LIF prepared in-house was added only when indicated.

### Immunofluorescent staining of ES cells and image quantification

Cells were fixed for 10 min with 4% PFA at room temperature, followed by permeabilisation and blocking in PBS containing 0.1% TritonX-100 and 3% donkey serum. Cells were incubated with primary antibodies (Table S7) in blocking solution overnight at 4°C. Alexa Fluor-conjugated donkey secondary antibodies (Molecular Probes) were used at 1:1000. For confocal microscopy imaging, ES cells were plated onto dishes with ibiTreat surface (Ibidi). Images were obtained using Leica SP5 confocal microscope for Fig. S2 and Leica 4000B standard fluorescent microscope for all other figures. Mean immunofluorescence (IF) and DAPI intensity per cell was quantified using Volocity (Fig. 3) and Cell Profiler (Fig. S2). For Fig. 3, cells were ordered according to increasing mean IF intensity in DAPI-positive particles and then consecutive 25, 50 and 25 percentiles of positive cells were labelled as low, mid and high for a particular marker.

### Monolayer differentiation, flow cytometry, cell sorting and clonogenicity assays

Cells were plated at 1.5×10^4^ cells cm^−2^ in 2i and medium was replaced with N2B27 or fresh 2i after 20-24 h. Prior to sorting, cells were washed, pelleted and resuspended in the respective culture medium. For dissociation of 2i cells, 2i inhibitors were added into Accutase. ToPro-3 (Invitrogen) was added at a concentration of 0.05 nM to label membrane-compromised cells. Cells were sorted in MoFlo flow sorter (Beckman Coulter). From 2i- and 16 h-cultures, all ToPro-3-negative cells were collected. To obtain Rex1-high and Rex1-low fractions from 25 h-cultures, sorting gates were set to collect cells with highest and lowest 15% GFP expression. For clonogenicity assays, 500 or 800 cells, as indicated in the figure legends, were plated on six-well dishes in Serum/L or 2i/L and coated with 0.1% gelatine or laminin (Sigma, L2020), respectively. On days 4 (Serum/L) or 6 (2i/L), plates were fixed and stained for AP (Sigma, 86R-1KT). Plates were scanned using a Cell Celector (Aviso) and scored manually. Colony formation efficiency for a given population was determined by dividing the average number of colonies formed in 2i/L by that in Serum/L. Flow cytometry was performed using a Dako Cytomation CyAn ADP high-performance cytometer and results were analysed using Summit.

### RNA extraction, cDNA synthesis and qPCR

Total RNA was isolated using RNeasy mini kit (Qiagen). cDNA was synthesised using SuperScript III (Invitrogen) and oligo-dT primers. qRT-PCR was performed with TaqMan Gene Expression assays (Thermo Scientific) using probes listed in Table S7.

### Single cell RT-qPCR

Cells were sorted using a G1 enrichment strategy, based on forward scatter (FS) and side scatter (SC) gating. Single cells were sorted into 96-well plates containing CellsDirect One-Step qRT-PCR master mix (Invitrogen, 11753-100) for cDNA synthesis and pre-amplification. Fluidigm assays were performed according to the manufacturer's protocols using EvaGreen detection at the Genomics Core Facility at the European Molecular Biology Laboratory in Heidelberg, Germany. Primer sets used are listed in Table S7.

### Protein analysis by mass spectrometry

Nuclear fractions from naïve ES cells and transiting populations were subjected to proteomic analysis by mass spectrometry using SILAC, as described in the supplementary Materials and Methods.

### Detection of OCR and ECAR by extracellular flux analysis

Extracellular flux analysis was carried out using the Mito Stress Assay in a Seahorse XFe24 Analyzer, according to the manufacturer's protocol (Agilent Technologies). See supplementary Materials and Methods for details.

### Cycloheximide treatment

ES cells were subjected to the standard differentiation protocol (Fig. 4A). Six hours after medium switch to 2i or N2B27, cycloheximide (Sigma, C4859) (100 µg/ml) or DMSO (control) was added into the media.

### Immunoblots

Western blots were performed on total cell lysates as described in the supplementary Materials and Methods.

### Mouse colony establishment and immunostaining of embryos

Mice carrying the Rex1-GFPd2 reporter were generated as described in the supplementary Materials and Methods.

### Microarray, RNA-sequencing, DNA methylome and proteome analyses

Processing of ES cell samples and data analyses are described in the supplementary Materials and Methods.
